# The *Escherichia coli* Outer Membrane β-Barrel Assembly Machinery (BAM) Crosstalks with the Divisome

**DOI:** 10.3390/ijms222212101

**Published:** 2021-11-09

**Authors:** Elisa Consoli, Joen Luirink, Tanneke den Blaauwen

**Affiliations:** 1 Bacterial Cell Biology and Physiology, Swammerdam Institute for Life Science, University of Amsterdam, 1098 XH Amsterdam, The Netherlands; e.consoli@uva.nl; 2 Department of Molecular Microbiology, Amsterdam Institute of Molecular and Life Sciences (AIMMS), Vrije Universiteit, De Boelelaan 1085, 1081 HV Amsterdam, The Netherlands; s.luirink@vu.nl

**Keywords:** *Escherichia coli*, β-barrel assembly machinery, BAM complex, divisome, Sec machinery, immunolabelling

## Abstract

The BAM is a macromolecular machine responsible for the folding and the insertion of integral proteins into the outer membrane of diderm Gram-negative bacteria. In *Escherichia coli*, it consists of a transmembrane β-barrel subunit, BamA, and four outer membrane lipoproteins (BamB-E). Using BAM-specific antibodies, in *E. coli* cells, the complex is shown to localize in the lateral wall in foci. The machinery was shown to be enriched at midcell with specific cell cycle timing. The inhibition of septation by aztreonam did not alter the BAM midcell localization substantially. Furthermore, the absence of late cell division proteins at midcell did not impact BAM timing or localization. These results imply that the BAM enrichment at the site of constriction does not require an active cell division machinery. Expression of the Tre1 toxin, which impairs the FtsZ filamentation and therefore midcell localization, resulted in the complete loss of BAM midcell enrichment. A similar effect was observed for YidC, which is involved in the membrane insertion of cell division proteins in the inner membrane. The presence of the Z-ring is needed for preseptal peptidoglycan (PG) synthesis. As BAM was shown to be embedded in the PG layer, it is possible that BAM is inserted preferentially simultaneously with de novo PG synthesis to facilitate the insertion of OMPs in the newly synthesized outer membrane.

## 1. Introduction

The cell envelope of Gram-negative bacteria is a tripartite structure that offers protection and resistance against external insults, such as detergents, toxins, and antibiotics [1]. This selective barrier is composed of an inner membrane (IM), a peptidoglycan (PG) layer, and an outer membrane (OM) [2]. The crowded aqueous space between the IM and the OM is called periplasm. The OM is the first line of bacterial defense: it prevents the intracellular access of antibiotics, partially accounting for why Gram-negative bacteria are generally insensitive to several common antibiotics that do act on Gram-positive bacterial species. 

The OM is an asymmetric bilayer composed of phospholipids in the inner leaflet and lipopolysaccharides (LPS) exclusively in the outer leaflet [3]. Due to the low permeability of the OM, Gram-negative bacteria have evolved systems to enable communication with the external milieu: the outer membrane proteins (OMPs). The OMPs share the same ‘β-barrel’ structure, formed by a variable number of antiparallel β-strands that create an inner barrel hydrophilic lumen for the transport of molecules. Like all the proteins, OMPs are synthesized in the cytoplasm, and they need to be transported to reach their destined location. They are synthesized with an N-terminal signal sequence, which drives targeting to, and translocation across, the inner membrane via the Sec translocon. In *Escherichia coli*, the nascent OMPs follow a SecB and SecA dependent post-translational targeting mechanism and use the SecYEG translocon to cross the IM [4,5]. 

The Sec translocon consists of the heterotrimeric channel complex SecY, SecE and SecG that acts together with accessory components, such as the chaperone/insertase YidC [6,7]. Proteins are secreted in a vectorial fashion from the amino- to the carboxy-terminus like ‘spaghetti through a hole’ [3]. Once the OMPs reach the periplasm, the signal sequence is cleaved off by signal peptidase I, releasing the proteins from the membrane [8,9]. To keep them in a folding competent state, they recruit periplasmic chaperones and holdases. The unfolded OMPs are then delivered by chaperones at the β-barrel assembly machinery (BAM), which recognizes, folds, and inserts the β-barrels into the OM [10]. 

The BAM complex is well conserved amongst the Gram-negative bacteria. In *E. coli*, it is composed of five subunits (BamA-E), in a 1:1:1:1:1 stoichiometry [11]. BamA is the essential core of the machinery. It is a β-barrel itself that forms the protein–lipid interface, and it contains a periplasmic domain composed of five extensible POTRA (polypeptide transport-associated) repeats. The other four subunits are periplasmic lipoproteins attached to the OM by their lipid moiety on the amino-terminus and anchored by non-covalent interactions to each other and/or with BamA. The BamB subunit forms an eight-bladed β-propeller that interacts directly with BamA. BamB is not essential, but its absence produces a strong OMP biogenesis defect [12,13,14,15]. The role of the non-essential BamC has not been elucidated yet, but it was recently found to be surface-exposed [16]. BamD is an essential subunit responsible for substrate recognition [17], and it is composed of a tandem of TPR (tetratricopeptide repeats) motifs. BamE is the smallest and non-essential subunit of the complex. BamB, BamC, BamD and BamE form a module suggested to drive a conformational switch in the BAM complex that enables β-barrel insertion into the OM [18]. The BAM complex plays a pivotal role in the maintenance of the OM integrity, exemplified by the essential BAM substrate, the β-barrel LptD, responsible for the insertion of LPS molecules into the outer layer of the OM [19]. 

Although much is known about the structure of the BAM complex, its in vivo cellular distribution has been studied only partially [20,21]. We have recently used an antiserum against the whole BAM complex to investigate BAM localization by whole-cell immunofluorescence [22] and showed that the PG layer is anchored by BAM. In the present work, we show that the BAM complex localizes in the lateral wall and at midcell in constricting cells. In *E. coli*, the divisome is a macromolecular machine that contains over 20 different types of proteins [23]. It coordinates the septal PG synthesis with IM and OM invagination, leading to the separation of two daughter cells. The assembly of the divisome machinery occurs in two discrete steps [24]. The first step involves the midcell occurrence of the bacterial tubulin-homologue FtsZ, with the help of other ‘early’ proteins, such as FtsA, ZipA and FtsK, at the future division site [25,26]. FtsZ monomers polymerize into short filaments to form the Z-ring, essential to template the cell division machinery [27]. In the second stage, the downstream ‘late’ components FtsB, FtsL, FtsQ, FtsW, PBP3 and FtsN join interdependently [28] to form the mature divisome. The arrival of FtsN, at the division site, is the crucial step to accomplish divisome core assembly and trigger septal PG synthesis to generate the new poles of the daughter cells [29,30,31]. Once the divisome complex is mature, the nascent septal PG is synthesized by the PBP3FtsW complex, possibly in combination with class A PBPs [32,33,34]. PBP3 is the main transpeptidase responsible for cell division [35,36,37]. Here, we investigate which protein is responsible for BAM midcell recruitment. By inhibiting septal peptidoglycan synthesis with aztreonam, we demonstrate that the BAM enrichment at the constriction site is not dependent on the active divisome. Then, we tested the localization of the complex in different thermosensitive division protein mutants. The BAM is still present at midcell in the absence of the late cell division proteins FtsQ, PBP3 and FtsN, at the constriction site. In contrast, the BAM midcell localization is lost upon the inhibition of the Z-ring formation by the FtsZ-specific toxin Tre1, suggesting that BAM complex recruitment at the division site relies on Z-ring formation. Moreover, we show that YidC localizes at midcell with a similar cell division timing as BAM, and its midcell localization is also dependent on the presence of FtsZ. YidC, in combination with SecYEG, is required for the insertion of divisome proteins [38,39,40]. The presence of Bam and the Sec machinery likely functions to facilitate insertion of proteins in the envelope of the newly synthesized cell poles. 

## 2. Results

### 2.1. The Bam Complex Localizes at Midcell during Constriction

**Figure 1:**
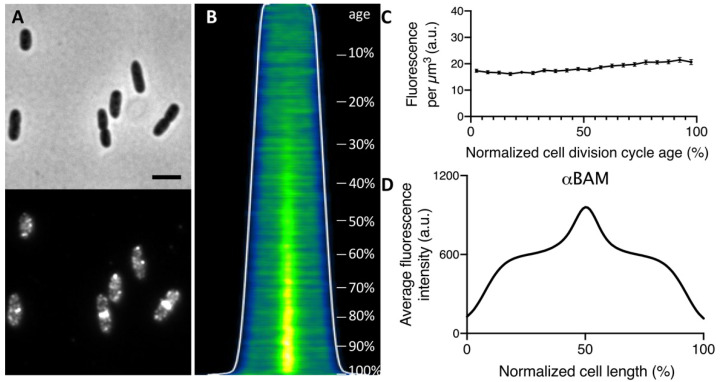
*E. coli* wild type cells LMC500 immunolabelled with polyclonal antibodies against BAM (αBAM). (**A**) Phase contrast and fluorescence microscopy images show the wild type grown to steady state in minimal medium at 28 °C and immunolabelled for the Bam complex. Scale bar equals 2 μm. (**B**) In the demographic map, the cells are sorted by cell length and the cell division cycle age is indicated (%). The longer/older cells clearly show BAM midcell localization from 40% onwards. (**C**) Concentration of the BAM fluorescence signal plotted as function of the normalized cell division cycle age shows an almost constant BAM production. The fluorescence concentration markers are the values of 5% age bins with the 95% confidence range indicated by the error bars. (**D**) The average profile of the fluorescence intensity plotted as function of the normalized cell length exhibits a peak at 50%, in correspondence of midcell. Antibodies against the BAM were all used 1:500 in cells permeabilized with Triton X-100 and lysozyme. The number of analyzed cells is 6770.

In order to observe the endogenous expression of the BAM complex, a polyclonal antiserum raised against the whole BamA-E complex, which recognizes all subunits (αBAM), was used for the immunolabeling of wild type *E. coli* K12 cells [22]. The immunofluorescence, performed on cells grown to steady state, revealed that the BAM complex displays a distribution along the cell surface in bright distinct foci (Figure 1A,B), consistent with previous studies [20,21,22]. Steady-state growth allows the correlation of cell length to cell age [41], enabling observation of the protein expression and localization over the course of the cell cycle. Analysis of the amount of fluorescence of the labelled BAM per cell divided by the calculated cell volume assuming the cell to have a rod-shape provides the BAM concentration per μm^3^. The BAM complex is continuously produced as its concentration remained quite constant at all cell ages when grown in minimal medium (Figure 1C), as well as in rich medium [22]. Strikingly, the fluorescence demographic map of cells sorted by their length revealed a BAM complex enriched at the site of constriction, in the second half of the cell division cycle (Figure 1B). This enrichment is further confirmed by the plot of the average fluorescence along the normalized cell length, showing an intense BAM signal at 50% (Figure 1D). The midcell localization of the BAM complex was then measured for each cell by determining the extra fluorescence at the cell center (±0.2 μm) in comparison to the fluorescence in the rest of the cell. The complex started to accumulate at midcell around 40% of the cell cycle. In conclusion, the immunolabelling results in a BAM localization pattern of discrete foci along the cell axis, with a clear midcell localization starting from 40% of the cell division cycle time.

### 2.2. All BAM Subunits Localize at Midcell

**Figure 2:**
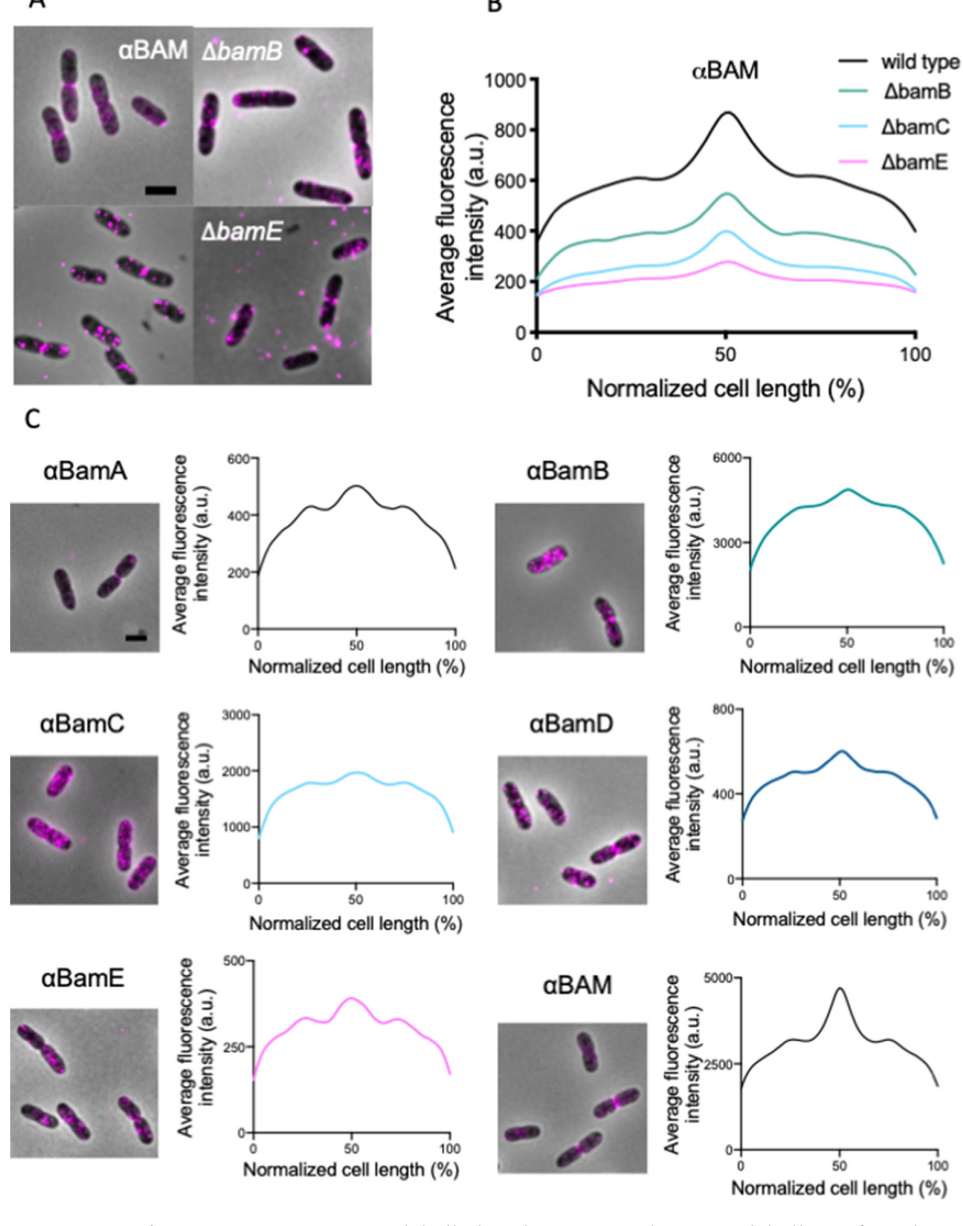
*E. coli* BAM mutants immunolabelled with αBAM and immunolabelling of single BAM subunits (αBamA-E). (**A**) Fluorescence microscopy images of BW25113 wild type and mutant cells grown in rich medium at 37 °C and immunolabelled for the BAM complex. (**B**) The average fluorescence profiles reveal a BAM midcell localization, for all the deletion mutants, with different degrees of intensity. (**C**) Microscopy images and average fluorescence profiles plotted as function of the normalized cell length of wild type cells immunolabelled against the single Bam subunits and αBAM. All the profiles exhibit a peak at midcell. The antibodies against the single BAM subunits likely exhibit a different affinity for their target explaining the variety in fluorescence intensities, but in each case, midcell localization was clearly observed. αBAM n = 1692; Δ*bamB* n = 1042; Δ*bamC* n = 1634; Δ*bamE* n = 1598; αBamA n = 2042; αBamB n = 1450; αBamC n = 2373; αBamD n = 2029; αBamE n = 1654. Scale bar is equal to 2 μm.

To investigate whether one of the non-essential BAM subunits is required for midcell localization, we immunolabeled strains that lack either BamB, BamC or BamE. From the data analysis, it is evident that the BAM midcell localization is occurring in all three strains (Figure 2A,B). Because the αBAM antibodies do not discriminate between the individual subunits, specific antibodies against each subunit were used to confirm the presence of all individual subunits at midcell. All the average profiles, obtained by the immunofluorescence of single BAM subunits, show a similar localization pattern: foci distribution along the cell axis and an enhanced signal at midcell (Figure 2C). 

**Figure 3:**
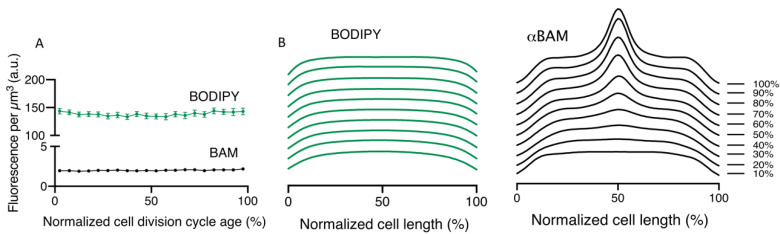
Midcell occurrence of BAM and Bodipy C-12. (**A**) LMC500 wild type cells grown to steady state at 28 °C, stained with the membrane staining Bodipy-C12 (green) and with antibodies against the BAM (black). The concentration of the fluorescent signals of both the membrane (green) and the BAM (black), plotted as function of the normalized cell division cycle, show a constant concentration over time. (**B**) The fluorescence profiles were binned into 10 age classes (10% to 100%) and plotted as function of the normalized cell length. The BAM complex exhibits an enrichment at midcell, in correspondence to 40% of the cell division cycle age. The membrane staining does not change in distribution during time. BAM and Bodipy-C12 (n = 1956).

To address the potential caveat that this midcell occurrence is erroneous, due to the putative higher membrane content during constriction, the BAM signal was compared to a general membrane staining. The wild-type strain was grown to steady state, fixed, immunolabelled with αBAM, and stained with the lipid dye Bodipy-C12. Both the BAM and the Bodipy-C12 signal show a constant concentration of fluorescence as a function of the cell division cycle age (Figure 3A). However, when the BAM and the lipid dye fluorescence average cell profiles were binned in 10 different age classes (10–100%), BAM shows clear midcell localization, that becomes visible around 40% of the cell division cycle age, while the Bodipy-C12 does not (Figure 3B). This result confirms the specificity of the αBAM midcell fluorescent signal. In conclusion, in *E. coli*, immunolabelling confirms that all BAM subunits accumulate at the center of the cell during septation, probably reflecting midcell localization of the entire assembled BAM complex.

### 2.3. Not All OM Proteins Exhibit a Midcell Localization like BAM

**Figure 4:**
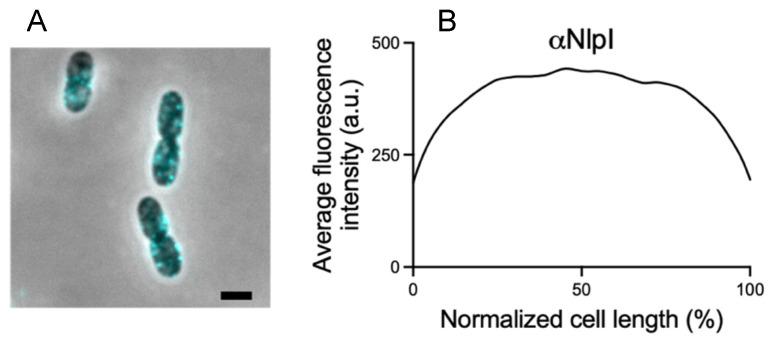
*E. coli* wild type cells immunolabelled against NlpI. Microscopy images (**A**) and fluorescence average profile (**B**) show no evident midcell localization for the NlpI lipoprotein. LMC500 wild type cells were grown to steady state in minimal medium at 28 °C. (n = 1015). Scale bar is equal to 1 μm.

We demonstrated that the BAM enrichment at midcell is not due to a higher membrane content during constriction. To gain further insight into the specificity of BAM recruitment at midcell, we investigated the localization pattern of the unrelated outer membrane lipoprotein NlpI during the cell cycle. Immunolabelling of the steady state grown wild type cells with polyclonal antibodies specific for the OM lipoprotein NlpI [42] revealed localization in bright foci along the cell surface without any specific midcell accumulation (Figure 4). We conclude that not all the OM proteins share the midcell localization of BAM during constriction.

**Figure 5:**
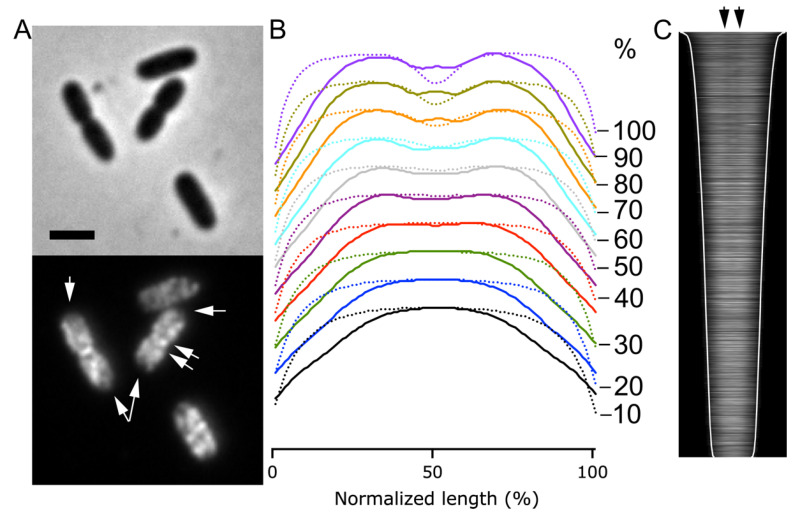
*E. coli* wild type cells immunolabelled against OmpA. LMC500 wild type cells were grown to steady state in minimal medium at 28 °C. (**A**) Phase contrast image on top, and the corresponding fluorescence image at the bottom, with arrows indicating the empty old poles and the empty new poles adjacent to the leading edge of the constriction. Scale bar is equal to 2 μm. (**B**) Fluorescence average profiles in age bins of 10%, as indicated. The dotted line presents the diameter of the cells, and the solid line, the OmpA fluorescence. (**C**) Demograph of the fluorescence of OmpA of all cells sorted according to their cell length, and with the edge of the cells indicated by the white line. The black arrows indicate the part of newly synthesized cell poles that do not have OmpA (n = 3223).

OMPs are reported to be inserted by BAM and therefore likely to be also present at midcell, as was reported previously [21]. To verify whether this is indeed the case, the OM β-barrel protein OmpA was immunolabelled. The antibodies against OmpA did not label an OmpA deletion strain and were therefore considered to be specific (Supplementary Figure S1). Interestingly, OmpA appeared to localize in the lateral wall, but not in the cell poles of young cells (Figure 5A). In dividing cells, OmpA is present in the newly synthesized cell pole, but only at the leading edge of the PG synthesis. In the older parts of the new cell pole, it is absent, as indicated by the arrows at the constriction site in Figure 5A, in the average fluorescence profiles in age bins of 10% (Figure 5B) and in the demographic maps of the fluorescence sorted according to length (Figure 5C). The results suggest that OmpA is inserted during septal PG synthesis and then disappears by lateral diffusion or enzymatic degradation. In conclusion, the topography of BAM in the cells is specific, as other OM proteins such as NlpI and OmpA clearly have a different distribution.

### 2.4. The BAM Complex Localizes at Midcell Simultaneously with Divisome Maturation

**Figure 6:**
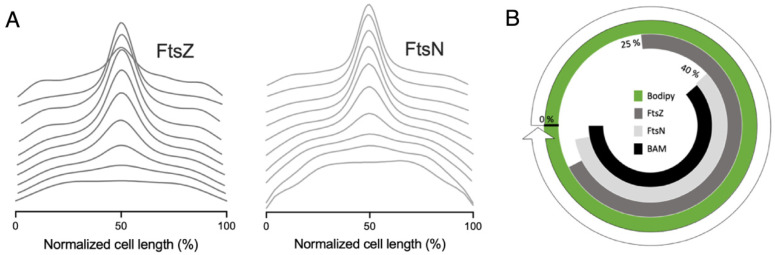
Midcell occurrence of FtsZ, FtsN. (**A**) LMC500 wild type cells grown to steady state in minimal medium at 28 °C and immunolabelled against the division proteins FtsZ (dark grey) and FtsN (light grey). The FtsZ and FtsN fluorescence profiles were binned into 10 age classes (10% to 100%) and plotted as function of the normalized cell length. From the midcell enrichment, the timing of FtsZ and FtsN during the cell division cycle can be observed. FtsZ (n = 3300); FtsN (n = 5921). (**B**) The presence at midcell, represented as a circular timeline map, offers the timing of the divisome assembly: it starts at 25% with the accumulation at midcell of the ‘early’ protein FtsZ and ends with the localization at midcell of the ‘late’ division protein FtsN, around the 40% of the normalized cell division cycle age. BAM and FtsN are recruited simultaneously at midcell.

The cell division assembly occurs in two steps with a defined timing. To test whether there is any correlation between the divisome assembly and the localization of the BAM machinery, we compared the midcell occurrence of these two consecutive complexes. Wild type cells grown to steady state were immunolabelled for FtsZ and FtsN, i.e., the first and the last protein that assemble at the division site, respectively (Figure 6A). We also immunolabelled the cells for the BAM complex (Figure 3B). The extra intensity of fluorescence at midcell in comparison to the rest of the cell of each protein was measured to calculate the midcell arrival time, and from that information, a radial timeline map was built (Figure 6B). FtsZ and FtsN started to accumulate at 25% and 40% of the cell division cycle, respectively, which was directly followed by BAM. As reported [43], once FtsZ arrives at midcell, it continues to accumulate up to 60%, after which it remains constant until 90% of the cell cycle. FtsN, conversely, starts accumulating at the division site from 40%, until it reaches the maximum at 90% of the cell division cycle age, when FtsZ is already leaving [24]. In contrast, the BAM complex is increasing at midcell, becoming visible from 40%, and declining around 95% of the cell division cycle age. (Figure 6B). Our result suggests that the BAM localizes at the site of constriction simultaneously with the maturation of the divisome. This indicates that it is preferentially inserted in the new cell poles.

### 2.5. The BAM Midcell Localization Does Not Require Active Septation

**Figure 7:**
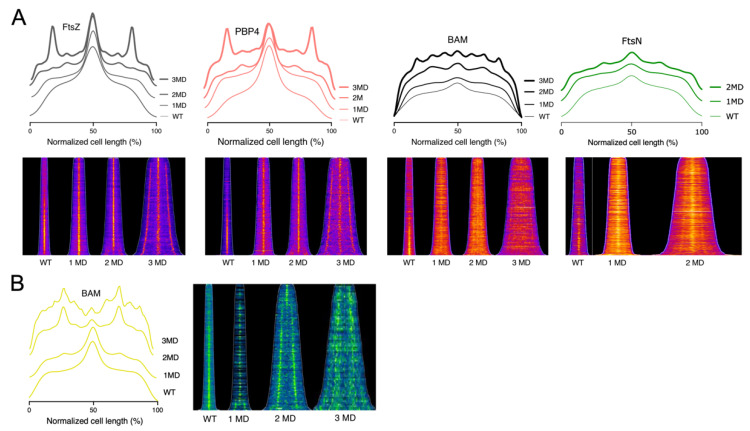
Localization of FtsZ, PBP4 and the BAM complex in LMC500 *E. coli* wild type cells treated with the PBP3 inhibitor aztreonam and in a thermosensitive PBP3 mutant. (**A**) The cells were grown to steady state in GB1 at 28 °C (WT) or diluted in prewarmed medium and incubated with aztreonam for 1, 2 and 3 mass doublings (1–3 MD), before harvesting and immunolabelling with antibodies against FtsZ, PBP4 BAM and FtsN. The fluorescence average profiles and the corresponding fluorescence demographic maps are shown in the top panel and lower panel, respectively. FtsZ, PBP4 and FtsN reflect the divisome stalling at 25, 50 and 75% of the cell length of the by aztreonam created filaments. The BAM complex follows the divisome localization, albeit with lower intensity. Numbers of cells analyzed were for αFtsZ 5047, 1433, 858 and 660 for WT, 1MD, 2MD and 3MD, respectively. Numbers of cells analyzed were for αPBP4 2347, 1856, 1638 and 615 for WT, 1MD, 2MD and 3MD, respectively. Number of cells analyzed were for αBAM 3036, 1980, 1160 and 747 for WT, 1MD, 2MD and 3MD, respectively. Number of cells analyzed were for αFtsN 984, 711 and 610 for WT, 1MD, and 2MD, respectively. (**B**) The average fluorescence profiles of BAM in a PBP3ts mutant at permissive condition at 28 °C (WT), and at non-permissive condition at 42 °C for 1, 2 and 3 mass doubling (1, 2 and 3 MD). The BAM complex localizes at midcell, and at the future sites of constriction at 25% and 75% of the cell length. The corresponding fluorescence demographic maps of the immunolabelled BAM complex in these cells are shown on the right. Number of cells analyzed were 2378, 532, 529 and 193 for WT, 1MD, 2MD and 3 MD, respectively.

To investigate whether the BAM recruitment at the division site requires the presence of active septal peptidoglycan production, BAM localization was analyzed in cells grown in the presence of the PBP3 inhibitor aztreonam. Aztreonam inhibits PBP3, thus blocking septal PG biogenesis, without affecting divisome assembly [44,45]. Importantly, it was already shown that the aztreonam does not affect the overall level of BAM in the OM [21]. Incubating the bacteria with 1 μg·mL^−1^ of aztreonam, for 1, 2 or 3 generations, causes the cells to elongate without division. The divisome is stalled at midcell, while its assembly at the future division sites at 25% and 75% of the normalized cell length continues [45]. Wild type cells grown to steady state were incubated with or without aztreonam; up to 3 mass doublings (MDs). Subsequently, the cells were immunolabelled with αFtsZ, αPBP4, αFtsN and αBAM. PBP4 is known to localize simultaneously with FtsZ at midcell during constriction [46]. In cells treated with aztreonam, the localization of FtsZ, FtsN and PBP4 reflected the divisome assembly at midcell, and at ¼ and ¾ of the cell length (Figure 7A and Supplementary Figure S2). Clearly, under these conditions, the BAM complex still localized at potential division sites, as was reported for most cell division proteins, although it became less pronounced (Figure 7A). This result implies that at least part of the BAM accumulation at the division site does not require active peptidoglycan synthesis by the FtsW/PBP3 complex.

### 2.6. The BAM Midcell Localization Does Not Depend on the Late Division Proteins

The BAM midcell localization does not require an active septal PG synthesis; therefore, we decided to test whether this occurrence is dependent on the complete and mature divisome. To answer this question, we immunolabelled a thermosensitive (ts) PBP3 mutant *ftsI2158*(ts) [47] for BAM. PBP3 is an essential divisome transpeptidase that is recruited relatively late to the divisome, and in turn, responsible for the recruitment of FtsN [48]. At 42 °C, the PBP3 protein is unstable and degraded [32], leading to an incomplete divisome, which results in cell division arrest. Being one of the filamentous temperature sensitive (*fts*) genes, its absence results in filamentous cell morphology [48]. The BAM complex shows normal midcell localization when the PBP3ts mutant was grown at permissive conditions (Figure 7B). From the immunofluorescence average profiles of mutant cells, grown at the non-permissive condition for 3 MDs, we can observe a peak at the center, and at ¼ and ¾ of the filament length (Figure 7B and Supplementary Figure S3). This indicates that BAM midcell localization is not dependent on the late divisome proteins PBP3 and FtsN. Next, we tested another thermosensitive mutant of an upstream late cell division protein: *ftsQ1*(ts). After 2MDs at 42 °C, the BAM midcell localization is not affected, suggesting that the BAM complex does not require FtsQ for midcell localization (Supplementary Figure S4). Taken together, these results suggest that the BAM recruitment does not require complete assembly of the divisome. 

### 2.7. The BAM Complex Midcell Localization Depends on the Z-Ring Formation

**Figure 8:**
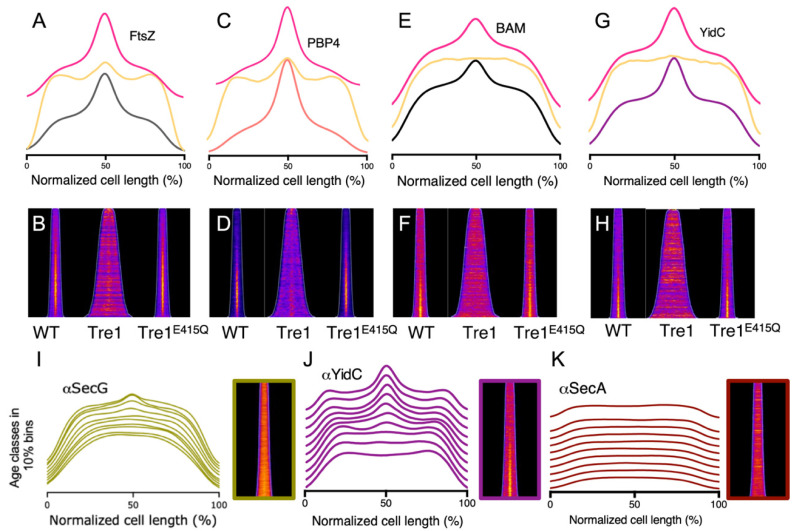
Localization of FtsZ, PBP4, YidC and the BAM complex in LMC500 *E. coli* wild type cells treated with Tre1 toxin FtsZ inhibitor and wild type *E. coli* cells immunolabelled for the Sec machinery. The cells were harvested after being grown to steady state in minimal medium at 28 °C (WT), or after expressing the plasmid encoded the Tre1 toxin (Tre) or its inactive variant (Tre1^E415Q^) for a further two mass doubling induced by 0.15% arabinose. (**A**,**C**,**E**,**G**) Fluorescence average profiles of FtsZ, PBP4, BAM and YidC of wild type cells (gray, orange, black and purple line, respectively), after the exposure to Tre1 toxin (yellow line) or to its inactive version (magenta line). (**B**,**D**,**F**,**H**) Fluorescence demographic maps of FtsZ, PBP4, BAM and YidC. Midcell Z-ring formation is impaired in cells expressing the Tre1 toxin (**A**,**B**). PBP4 midcell recruitment is strongly compromised by Tre1 expression (**C**,**D**). The BAM complex is evenly distributed along the cell envelope, upon Tre1 toxin induction. IM insertase YidC midcell presence is also completely abolished without the Z-ring formation. Number of cells analyzed were for αFtsZ 3477, 1100 and 3670 for WT, Tre1 and Tre1^E415Q^, respectively, for αPBP4 2392, 1607 and 3769 for WT, Tre1 and Tre1^E415Q^, respectively, for αBAM 2971, 1250 and 2563 for WT, Tre1 and Tre1^E415Q^, respectively and for αYidC 2698, 585 and 1998 for WT, Tre1 and Tre1^E415Q^, respectively. (**I**,**J**,**K**) Wild-type *E. coli* cells were immunolabelled for the Sec machinery components SecG, YidC and SecA. The fluorescence profiles were binned into 10 age classes (10% to 100%) and plotted as function of the normalized cell length. In both binned profiles, the IM subunit YidC exhibits a midcell accumulation around 40% of the cell division cycle. On the contrary, SecA binned profiles do not show any midcell enrichment, whereas SecG is only present in deeply constricting cells. The numbers of cells analyzed were 5329, 6979 and 3654 for αSecG, αYidC and αSecA, respectively.

The BAM midcell localization occurs simultaneously with the divisome assembly but does not depend on the late division proteins. To assess whether BAM localization depends on the early localizing divisome proteins, its midcell enrichment was tested in the absence of FtsZ polymerization. To prevent Z-ring formation, an antibacterial toxin was used, that is naturally expressed by the Gram-negative bacterium *Serratia proteomaculans*. The toxin, known as Tre1, targets the cell division process, through ADP-ribosylation of the amino acid residue R174 of FtsZ, preventing polymerization and hence Z-ring formation [49,50]. In *E. coli* wild type cells grown to steady state in minimal medium at 28 °C, Tre1 toxin was expressed from plasmid using an arabinose inducible promoter for 2 MDs. As a negative control, a catalytic inactive version of Tre1 toxin was used, in which the glutamic acid of the active site was substituted with glutamine (Tre1E415Q). After the expression, only the cells carrying the original variant of Tre1 toxin started filamenting, due to the impaired cell division process. To confirm that the Tre1 protein was causing the inhibition of FtsZ polymerization at midcell, the cells were immunolabelled with αFtsZ antibodies. From the fluorescence average profiles and from the fluorescence demographic maps, it is evident that the Z-ring is not assembled and the FtsZ signal is dispersed along the cell (Figure 8A,B and Supplementary Figure S5). We also immunolabelled PBP4, of which localization was shown to be dependent on FtsZ localization [46]. As expected, PBP4 midcell fluorescent signal was also lost in cells expressing Tre1 toxin (Figure 8C,D). From BAM immunolabeling analysis in Tre1 treated cells, it is evident that BAM is also distributed homogeneously along the cell axis rather than being enriched at the division site (Figure 8E,F). These results suggest that the BAM complex occurrence at the division site is strictly dependent on the Z-ring formation.

### 2.8. YidC Colocalize at Midcell at the Same Time as the BAM Complex

OMPs are produced in the cytoplasm and cross the IM via the SecYEG translocon, where periplasmic chaperones are engaged to deliver them to the BAM complex. The midcell localization of BAM could reflect its ‘hotspot’ activity. During septation, the nascent cell poles might require the BAM complex to locally fold and insert ex novo OMPs into the newly synthesized OM. This activity could then also require a local increase abundance of the SecYEG translocon. To verify this hypothesis, *E. coli* wild type cells were immunolabelled with specific antibodies against the Sec components SecG, SecA and the accessory chaperone/insertase YidC. From the binned fluorescence profiles, shown in Figure 8I–K (and Supplementary Figure S6), it is evident that YidC localizes at midcell around 40% of the cell division cycle age. SecA, on the contrary, exhibits an even distribution along the cell axis (Figure 8K), and SecG is only somewhat more abundant in the envelope of new cell poles of deeply constricting cells. A SecY-GFP fusion was also reported to be distributed evenly in the cell envelope [51]. Only YidC has a midcell localization trend that resembles the one from BAM. Notably, FtsEX and FtsQ are inserted via the SecYEG-YidC holotranslocon [38,39,40]. Although it is obvious that the Sec translocon is present at midcell, it does not seem to be enhanced with respect to the rest of the envelope. Possibly, YidC is specifically recruited to the SecYEG translocon at midcell because of its role in the insertion of divisome proteins into the inner membrane. Alternatively, the epitopes of YidC could be simply better accessible to antibodies. These results, taken together, could suggest a colocalization of the two complexes to ensure a more efficient OMPs transport. In this scenario, it is likely that, similar to BamA, the YidC midcell localization is also dependent on the Z-ring formation. To investigate this, YidC immunolabelling was performed on cells expressing Tre1 toxin. From the fluorescence profiles and maps (Figure 8G,H), it is evident that the YidC midcell accumulation is dependent on the polymerization of the FtsZ cell division protein. Such coordinated functionality could have evolved to insert rapidly new envelope material during the formation of the future daughter cell poles. 

## 3. Discussion

The Gram-negative bacteria, with their three-layered envelope, need to tightly coordinate cell growth and division. Several modular machineries crosstalk with each other to monitor the ongoing processes, and to ensure their correct localization and timing. In this study, we revealed the spatiotemporal topography of the BAM complex in *E. coli* cells, and its dependence on the presence of the Z-ring. The BAM complex localizes all along the cell periphery in distinct foci or clusters, as has been reported before [19,20,21,22]. Here, new insight was gained into the localization of BAM, by studying its cellular distribution during the cell division cycle. The BAM machinery was immunolabelled with antibodies generated against the isolated BAM complex or with antibodies against its single subunits and found to exhibit a striking enrichment at the division site. This occurs at a specific moment, that corresponds to 40% of the cell division cycle age. In wild type cells, the nascent divisome assembles with a cell cycle specific timing [24]. We used the first and the last protein FtsZ and FtsN that demarcate the beginning and the end of the divisome assembly, respectively, to observe whether BAM is localizing specifically during divisome maturation. The localization data indicate that BAM accumulates at midcell after the Z-ring is completed, and simultaneously with the localization of the divisome gatekeeper FtsN, which acts as a trigger that initiates the septation [52]. FtsN is also required for the recruitment of several proteins to the division site, such as amidases, or the membrane invagination trans-envelope complex Tol-Pal [53,54]. BAM could also belong to the machineries that are directly recruited by the divisome or by its activity. In this study, we demonstrated that the BAM midcell occurrence does not require an active synthesis of septal peptidoglycan. In fact, preventing septation by using the PBP3-inhibitor aztreonam resulted in the localization of FtsZ, BAM and PBP4 at midcell, and at the future division sites at ¼ and ¾ position of the filamentous cells. FtsN follows this pattern, indicating that the divisome is complete, while the TPase activity of PBP3 is inhibited. However, BAM as well as FtsN seem to increase their presence in the lateral wall upon inhibition of PBP3, which might indicate that the amount of local PG synthesis correlates with the amount of localized protein.

In a non-localizing PBP3 thermosensitive mutant, BAM is still present at the center of the cells and at the future division sites, suggesting that its localization is independent of late cell division proteins such as FtsN. In contrast, the BAM midcell localization was shown to require the early cell division protein FtsZ. Inhibiting the Z-ring formation, through the expression of the bactericidal Tre1 toxin [49], abolished midcell localization of the BAM complex. This suggests that the proto-ring formation and perhaps preseptal PG synthesis is a necessary requirement to ensure the BAM recruitment/insertion at midcell during constriction. 

OMP-biogenesis is believed to be midcell-biased [21,55]. This could be dictated by different possible reasons, like the substrate availability, local differences in PG composition due to the preseptal PG synthesis [56], local variation in the OM curvature or lipid composition, or the capability to interact with the Sec machinery. We analyzed the spatiotemporal behavior of some components of the inner membrane translocase system Sec machinery, to test whether this complex is potentially aligned with the BAM to efficiently insert OMPs in the nascent future cell poles. Interestingly, the accessory chaperone/insertase YidC follows the same midcell localization trend as BAM, while SecG was only more abundant in deeply constricting cells, whereas SecA was evenly distributed. Like BAM, YidC does not localize anymore at the site of constriction upon Tre1 toxin inhibition of Z-ring formation. YidC in complex with SecYEG is known to be involved in the membrane insertion of FtsQ [39,40]. Consequently, the Sec translocon will also be present at midcell and able to support the insertion of OMPs, as well as divisome proteins. 

As BAM seems to be embedded in the PG layer [22], it is possible that it can only be inserted at sites of active PG synthesis. During length growth, it would be inserted concomitantly with new strands of PG through elongasome activity, and during cell division, its enhanced presence is due to the increase in local PG synthesis by the divisome during septation. Inhibition of PBP3 by aztreonam causes a futile cycle of disaccharide polymerization by the glycosyltransferase FtsW, and the breakdown of this material by the soluble glycosyl hydrolases, such as Slt70 [57,58,59]. Consequently, aztreonam does not inhibit the localization of BAM at midcell. However, the absence of the late localizing proteins such as FtsQ, PBP3 and FtsN did not abolish BAM localization either, indicating that active septal PG synthesis is not a requirement for BAM midcell presence. Possibly, preseptal peptidoglycan synthesis is sufficient for BAM midcell localization, as it only requires the proto-ring, and not a mature divisome for localization. Preseptal PG synthesis depends on the presence of ZipA, FtsZ and PBP1A, but not PBP3 [56]. Due to the restricted mobility of OMPs in the OM [60,61], the coupling of PG synthesis to BAM OM insertion might help to ensure that areas of OM expansion also receive sufficient new OMPs.

In conclusion, these data suggest a more intimate relation between the OM BAM complex and the PG, in which BAM is inserted in the area where the PG is being synthesized. This mechanism will ensure the correct distribution of the OMP folding machinery in all areas of the cell that will need its presence the most. 

The bacterial cell envelope is still one of the main targets for the current discovery of alternative antimicrobials. Since the BAM complex is essential for the maintenance of the impermeability of the OM, knowledge of its specific function and biosynthesis might help to find inhibitors of the complex. This could either directly kill the bacteria or lead to an increased OM barrier permeability, allowing access to common antibiotics, that thus far kill exclusively Gram-positive bacteria. In addition, disrupting a mechanism that crosstalks with others, will lead to a ‘domino effect’ that will decrease the chances of developing multi-drug resistance.

## 4. Materials and Methods

### 4.1. Bacterial Strains and Culture Conditions

The *Escherichia coli* K12 Strains Used here are Listed in Table 1. 

The cells were cultured in minimal medium (6.33 g of K_2_HPO_4_ (Merck, Kenilworth, NJ, USA), 2.95 g of KH_2_PO_4_ (Riedel de Haen, Seelze, Germany), 1.05 g of (NH_4_)_2_SO_4_ (Sigma, St. Louis, MO, USA), 0.10 g of MgSO_4_·7H_2_O (Roth, Karlsruhe, Germany), 0.28 mg of FeSO_4_·7H_2_O (Sigma-Aldrich, St. Louis, MO, USA), 7.1 mg of Ca(NO_3_)_2_·4H_2_O (Sigma, St. Louis, MO, USA), 4 mg of thiamine (Sigma, St. Louis, MO, USA), 2 mg of uracil (Sigma, St. Louis, MO, USA), 2 mg of lysine (Sigma, St. Louis, MO, USA), 2 mg of thymine (Sigma, St. Louis, MO, USA), and 0.5% glucose (Merck, Kenilworth, NJ, USA) per liter, pH 7.0) for steady-state growth at 28 °C as described. Cells were cultured in rich medium (10g Tryptone (Bacto Laboratories, Mount Pritchard NSW, Australia), 5 g yeast extract (Duchefa, Amsterdam, The Netherlands) and 5 g NaCl (Merck, Kenilworth, NJ, USA) per liter) grown at 37 °C. Optical density (OD) was measured at 450 and 600 nm when grown in minimal medium and rich medium, respectively. To inhibit the PBP3 protein, *E. coli* LMC500 wild type cells were incubated with 1 μg⋅mL^−1^ aztreonam (ICN, Biomedicals OH, USA), previously dissolved in saturated Na_2_CO_3_. Temperature-sensitive mutants for the divisome proteins FtsI and FtsQ [47] were grown at 28 °C to OD450 of approximately 0.2. Cells were diluted in fresh minimal medium, pre-warmed at 28 and 42 °C, respectively, and kept growing for 3 mass doublings. To inhibit FtsZ, competent LMC500 cells were transformed with the plasmids pBAD18-Tre1 and pBAD-Tre1^E415Q^ [49]. The cells were grown in the presence of 50 μg⋅mL^−1^ ampicillin (Sigma-Aldrich, St. Louis, MO, USA) and 0.5% (*w*/*v*) glucose, then the expression of the Tre1 active and inactive form was obtained, replacing the glucose with 0.15% (*w*/*v*) arabinose.

### 4.2. Fixation and Immunolabelling

Cell populations were fixed with a mixture of 2.8% formaldehyde (Sigma-Aldrich, St. Louis, MO, USA) and 0.04% glutaraldehyde (Merck, Kenilworth, NJ, USA), keeping the culture shaking in the water-bath at growth temperature. After 15 min, the cells were harvested, and washed 3 times in Phosphate Buffered Saline (PBS, 0.2 g KCI, 0.2 g KH_2_PO_4_, 8 g NaCI, 2.16 g Na_2_HPO_4_·7H_2_O per liter of distilled water, pH 7.3) to remove the excess fixative. 

The fixed cell population was permeabilized with 0.1% Triton X-100 (Merck, Kenilworth, NJ, USA) in PBS (pH 7.2) at room temperature for 45 min, and 100 μg·mL*^−^*^1^ lysozyme (Sigma-Aldrich, St. Louis, MO, USA) and 5 mM EDTA (Sigma-Aldrich, St. Louis, MO, USA) for 45 min at room temperature, to permeabilize the membrane and the peptidoglycan, respectively [65]. By incubating the cells in 0.5% (*w*/*v*) blocking reagents (Boehringer, Mannheim, Germany) in PBS at 37 °C, the non-specific binding sites were blocked. Immunolabelling of the cells was performed with polyclonal rabbit antibodies αBAM, αBamA, αBamB, αBamC, αBamD, αBamE, αNlpI, αFtsZ, αFtsN, αPBP4, αSecG, αSecA (1:500) and αYidC (1:1000), dissolved in blocking reagent. The antibodies against the non-essential subunits BamB, BamC and BamE were previously pre-purified using the corresponding null mutant strain. The cells were further incubated with the donkey αRabbit IgG secondary antibodies conjugated with Cy3 or AlexaFluor488 (1:300, Jackson ImmunoResearch Laboratories, Inc., West Grove, PA, USA), according to the protocol [65].

### 4.3. Microscopy

The immunolabelled cells were immobilized on 1% agarose in water slabs coated object glasses as described [50] and imaged with a Hamamatsu ORCA-Flash-4.0 (Hamamatsu, Naka-ku, Japan) CMOS camera mounted on an Olympus BX-60 Fluorescence microscope (Tokyo, Japan) with a UPlanApo 100x/N.A. 1.35 oil Iris Ph3 objective. Images were acquired using the Micromanager 1.4 plugin for ImageJ (version 1.4, https://www.micro-manager.org, accessed on 4 November 2021) [66]. The fluorescence filter cubes used were U-MNG (Cy3 ex560/40, dic585LP, em630/75) and EN-GFP (AlexaFluor488, ex470/40, dic495LP, em525/50).

### 4.4. Image Analysis

Images were analyzed with Coli-Inspector, supported by the ObjectJ plugin for ImageJ (version 1.05, https://sils.fnwi.uva.nl/bcb/objectj/, accessed on 4 November 2021) [41]. Fluorescence and phase contrast images were aligned, and fluorescence background was subtracted as described [41]. The fluorescence of each cell was collected in a one-pixel wide bar with the length of the cell. A map of the fluorescence localization and intensity was generated with the cells sorted according to increasing cell length. Because cells were grown to steady state, the length of the cells can be directly correlated to the cell division cycle age [41]. A collective profile is created from all cell profiles in a map. They are first resampled to a normalized cell length of 100 data points, and then averaged to a single plot, in either 1 group or more age bins. The extra fluorescence at midcell in comparison to the fluorescence in the rest of the cell was calculated from the map of fluorescence profiles.

## Figures and Tables

**Table 1:** Strains and genotypes used in this study.

Strain	Genotype	Reference
BW25113	*lacI^q^ rrnB^T14^* Δ*lacZ_WJ16_ hsdR514* Δ*araBAD_AH33_* Δ*rhaBAD_LD78_*	[62]
BW25113Δ*ompA*	*lacI^q^ rrnB^T14^* Δ*lacZ_WJ16_ hsdR514* Δ*araBAD_AH33_* Δ*rhaBAD_LD78_ ompA::kan*	[62]
LMC500	*F- araD139* Δ*(argF-lac)U169 deoC1 flbB5301 ptsF25 rbsR relA1 rpsL150 lysA1*	[63]
Δ*bamB*	BW25113 Δ*bamB::cam*	[64]
Δ*bamC*	BW25113 Δ*bamC::cam*	[64]
Δ*bamE*	BW25113 Δ*bamE::cam*	[64]
PBP3*ts*	LMC500 *ftsI*(ts)	[47]
FtsQ*ts*	LMC500 *ftsQ*(ts)	[47]

## Data Availability

The data that support the findings of this study are available from the corresponding author upon reasonable request.

## Supplementary Materials

The following are available online at https://www.mdpi.com/article/10.3390/ijms222212101/s1.
