# Supplementary material for: The Escherichia coli Outer Membrane β-Barrel Assembly Machinery (BAM) Crosstalks with the Divisome
Source: Int J Mol Sci. 2021 Nov 9;22(22):12101. doi: 10.3390/ijms222212101 (PMC8620860; doi:10.3390/ijms222212101)
Supplement: Supplementary file 1 [file ijms-22-12101-s001.zip › ijms-1431266-supplementary.pdf]

International Journal of
Molecular Sciences

MDPI

Article

The *Escherichia coli* outer membrane β-Barrel Assembly machinery (BAM) cross-talks with the divisome

Elisa Consoli [1], Joen Luirink[2] and Tanneke den Blaauwen [1,*]

1 Bacterial Cell Biology and Physiology, Swammerdam Institute for Life Science, University of Amsterdam, 1098 XH Amsterdam, The Netherlands; e.consoli@uva.nl
2 Department of Molecular Microbiology, Amsterdam Institute of Molecular and Life Sciences (AIMMS), Vrije Universiteit, De Boelelaan 1085, 1081 HV, Amsterdam, The Netherlands
* Correspondence: t.denblaauwen@uva.nl

Abstract: The BAM is a macromolecular machinery responsible for the folding and the insertion of integral proteins into the outer membrane of diderm Gram-negative bacteria. In *Escherichia coli*, it consists of a transmembrane β-barrel subunit, BamA, and four outer membrane lipoproteins (BamB-E). Using BAM specific antibodies, in *E. coli* cells, the complex is shown to localize in the lateral wall in foci. The machinery was shown to be enriched at mid-cell with specific cell cycle timing. The inhibition of septation by aztreonam did not alter the BAM mid-cell localization substantially. Furthermore, the absence of late cell division proteins at mid-cell did not impact BAM timing nor localization. These results imply that the BAM enrichment at the site of constriction does not require an active cell division machinery. Expression of the Tre1 toxin, which impairs the FtsZ filamentation and therefore mid-cell localization, resulted in the complete loss of BAM mid-cell enrichment. A similar effect was observed for YidC that is involved in membrane insertion of cell division proteins in the inner membrane. The presence of the Z-ring is needed for preseptal peptidoglycan (PG) synthesis. As BAM was shown to be embedded in the PG layer, it is possible that BAM is inserted preferentially simultaneously with de novo PG synthesis to facilitate the insertion of OMPs in the newly synthesized outer membrane.

Keywords: ***Escherichia coli*, β-barrel assembly machinery, BAM complex, divisome, Sec machinery, immunolabelling.**

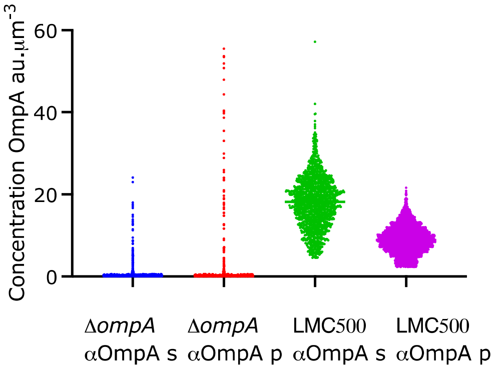

Figure S1. Antibodies against OmpA are specific. The cells were grown in rich medium at 37°C and immunolabelled with antibodies against OmpA. The concentration of OmpA fluorescence per µm³ cell volume is shown as violin plot. An *ompA* deletion strain was immunolabelled with antiserum against OmpA (red, n = 1581) and the supernatant was used to label another batch of these cells (blue, n = 1197). LMC500 was also labeled with the serum against OmpA (green, n =1562) and another batch was immunolabeled with the supernatant of the o*mpA* deletion strain (purple, n=2682). s =serum and p =pure (pre-adsorbed). The deletion strain does not show any specific labeling or adsorption of the antibodies as evidenced by only a slightly lower labeling density of the LMC500 cells due to dilution of the adsorbed serum.

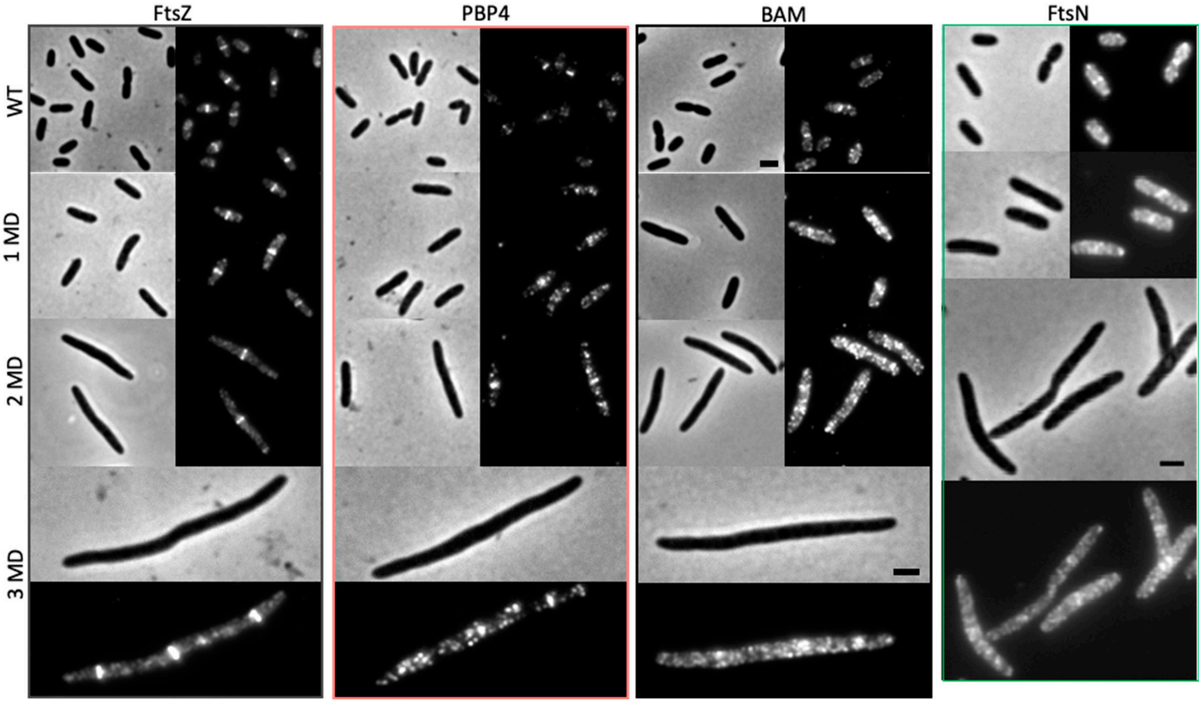

Figure S2. Immunolabelling of FtsZ, PBP4, the BAM complex and FtsN in *E. coli* LMC500 wild type cells treated with aztreonam PBP3 inhibitor. The cells were grown to steady state in GB1 at 28 °C (WT) and incubated with aztreonam for 1, 2 and 3 mass doublings (1-3 MD), then immunolabelled against FtsZ, PBP4, BAM and FtsN (1 and 2 MD). Phase contrast and fluorescence microscopy images reveal the cellular localization of the early division protein FtsZ, the division protein PBP4, FtsN and the β-barrel assembly machinery BAM. Scale bar equals 2 µm.

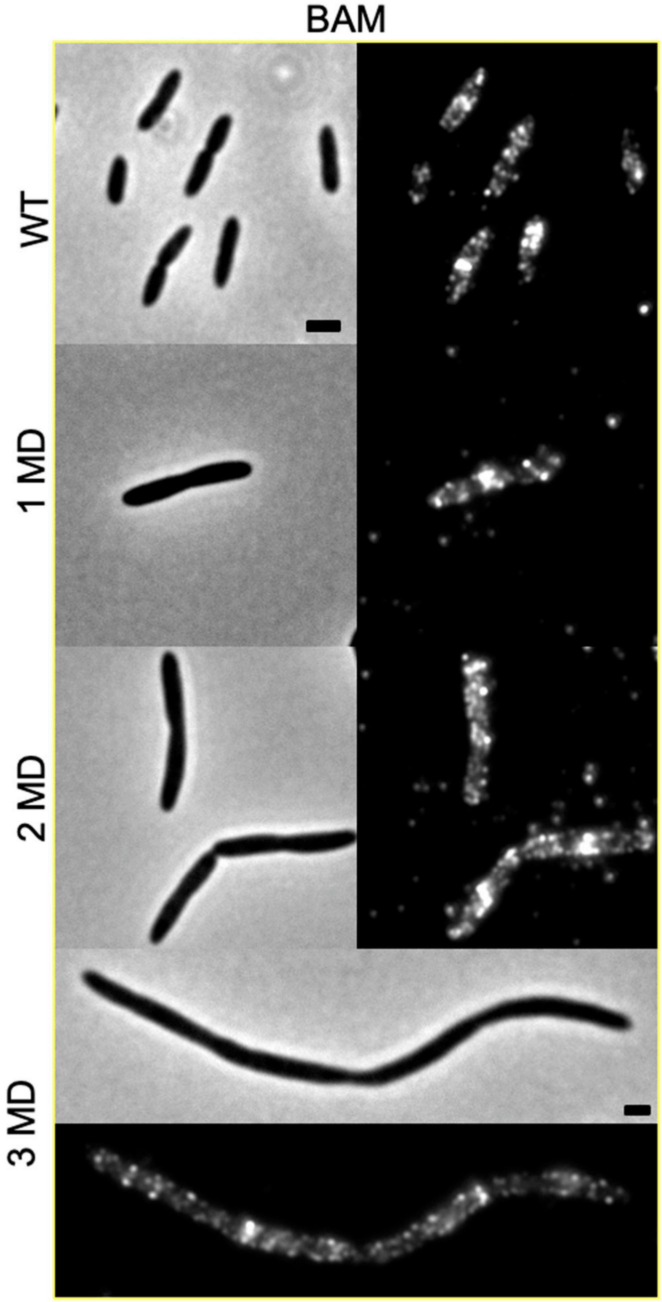

Figure S3. Immunolabelling of BAM in mutant *E. coli* thermosensitive for the cell division protein PBP3. Phase contrast and fluorescence microscopy images of immunolabelled BAM that shows localization in cells upon growth to steady state in GB1 at 28 °C (WT) and a subsequent shift to the non-permissive temperature of 42 °C. Scale bar equals 2 μm.

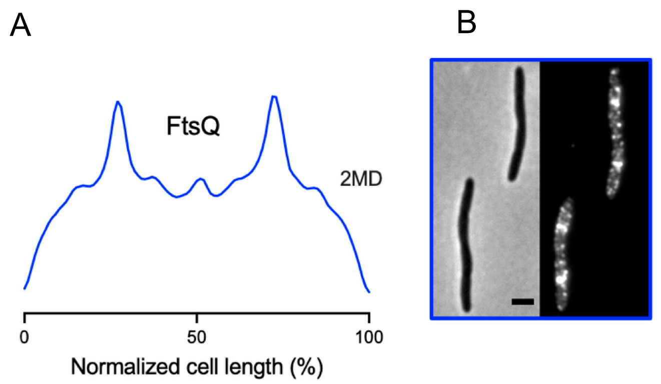

Figure S4. Immunolabelling of BAM in *E. coli* thermosensitive mutant for the cell division protein FtsQ. A, Fluorescence profile of the axial distribution of the BAM complex, after 2 mass doubling at the non-permissive temperature of 42°C (2MD). BAM still localizes at mid-cell and at the future division sites (25% and 75%). B, phase contrast and fluorescence microscopy image of BAM localization in cells grown to steady state and at non-permissive condition for 2 MD. Scale bar equals 2 µm.

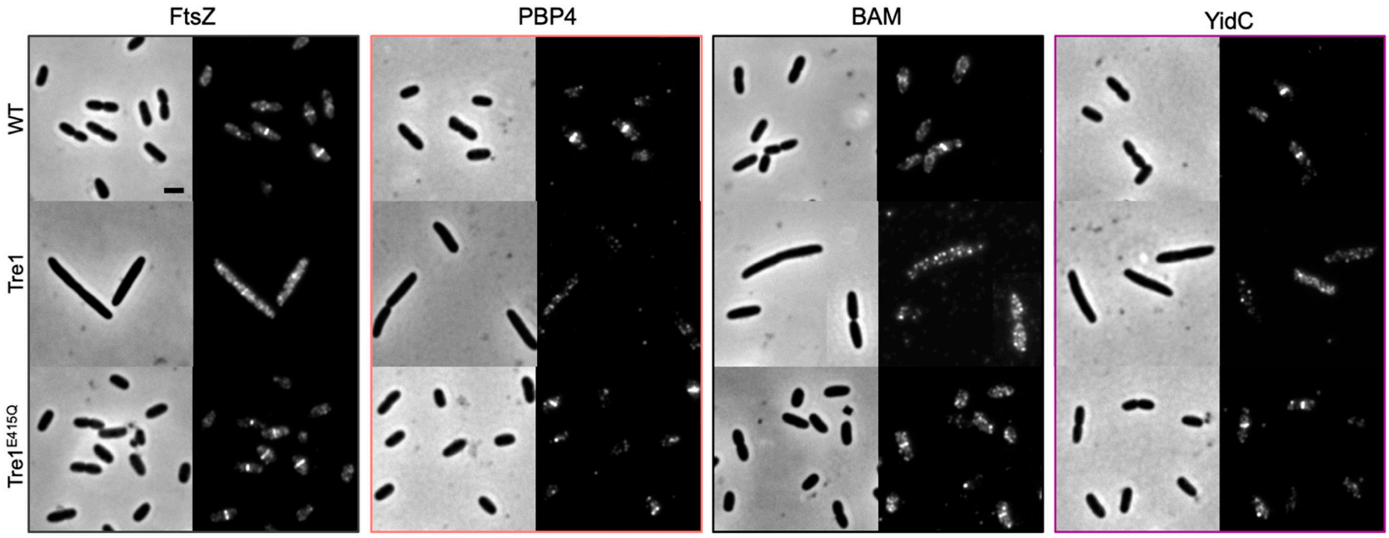

Figure S5. Immunolabelling of FtsZ, PBP4, YidC and the BAM complex in *E. coli* LMC500 wild type cells treated with Tre1 toxin FtsZ inhibitor. The cells were grown to steady state in minimal medium at 28 °C (WT) and a harvested or after subsequently expressing the plasmid encoded the Tre1 toxin (Tre) or its inactive variant (Tre1E415Q) for two mass doubling induced by 0.15% arabinose. Phase contrast and fluorescence images of cells immunolabelled against FtsZ, PBP4, BAM and YidC (gray, orange, black and purple line, respectively). Upon the Tre1 expression, the mid-cell accumulation of these proteins is strongly compromised. The BAM complex and the YidC localization at the constriction site is dependent on the presence of a Z-ring. Scale bar equals 2 µm.

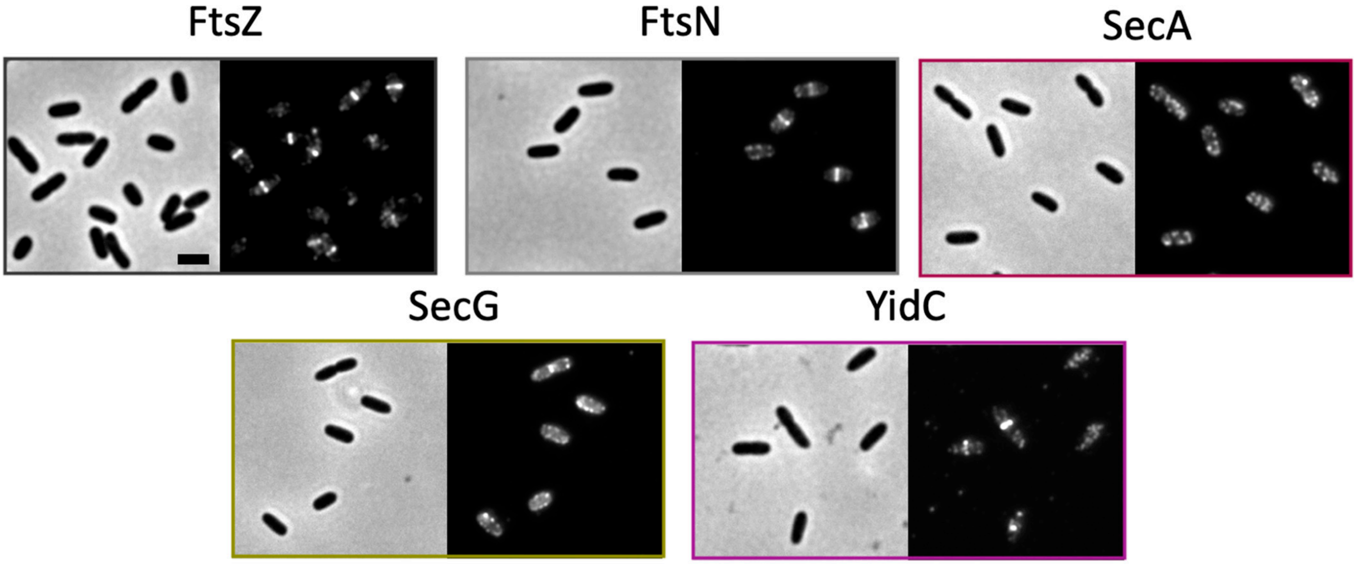

Figure S6. *E. coli* LMC500 wild type cells immunolabelled with polyclonal antibodies against divisome and IM translocon proteins. Phase contrast and fluorescent microscopy images of cells immunolabelled for the division protein FtsZ and FtsN (dark and light grey, respectively). Phase contrast and fluorescent microscopy images of cells immunolabelled for the IM translocon components SecA (burgundy), SecG (mustard) and YidC (purple). Scale bar equals 2 μm.
